# Acute Enteritis

**Published:** 1853-09

**Authors:** W. S. Linn

**Affiliations:** Chili, Ills.


### *Report of a case of Acute Enteritis.*

BY W. S. LINN, M. D., OF CHILI, ILLS.

Mary ———, aged 7 years, of scorbutic habit, was taken ill on the 19th of the past June. A medical attendant of the vicinity was called, who pronounced the morbid dificulty to be—parasites in the alimentary canal. His treatment founded on this diagnosis, consisted of purgative anthelmintics, and drastic cathartics, with exhibitions of large portions of sulph. morphiæ. Few worms, however, were expelled by this heroic treatment; and with time, her symptoms became proportionately exasperated. Her former attendant continued to visit her for four or five days, during which period the child grew hourly worse. At the expiration of this time, he rendered the opinion, that the child was convalescent, and refused to comply with a subsequent summons to visit her, saying that she was "quite well," and that his attentions were no longer required. At length, my attendance being desired, the following was found on the next day to be her condition: Pulse 160, respiration 40 in the minute, vigorous pyrexia, burning heat, exquisite tenderness over the entire extent of the abdominal parietes, skin dry and constricted, thighs firmly flexed on the trunk, tongue pointed, morbidly red and loaded with a dry bilious coating, frequent stools of a liquid character, suspending numerous groups of mucous flocculi, great restlessness and jactitation, anxiety strongly depicted in the countenance, and cerebral symptoms, manifested by a low, plaintive and piteous wail. With all the phogosis of the intestines and the high sthenic action, the stomach did not by vomiting or particular tenderness in that locality, evince its implication in the catalogue of morbid phenomena, but the sympathy between the brain and the tissues involved, was sufficiently obvious to warn the observer of their immediate relations.

The first night, the following course was adopted: Six powders composed of Hydrarg. Sub. mur., Doveri, Ipecac, and Sodæ Bicarb, were administered, one every three hours; warm mustard pediluvia, tepid sponging to the whole surface; head occasionally to be wet with cold water; cold applications strictly ordered to the bowels. The second day found the patient somewhat improved; the liquid, colorless dejections replaced by those of a bilious nature; fever less, thirst not so great, skin slightly relaxed, yet with acute sensibility to pain on pressure over the abdomen. The bowels appearing thoroughly cool at this time, I ordered a large epispastic, which produced free vesication over almost the entire abdominal region. The blister gave great relief. Prescriptions similar in effect to the foregoing were directed, from day to day; the blister was dressed with stimulating applications. At the end of four days, it become evident that a total subsidence of the inflammatory action had not taken place, and the counter-irritation was again resorted to, and the opiate treatment dismissed in consequence of augmenting cerebral sympathy, which began to give rise to symptoms of functional derangement, as prominent as the visceral primarily assailed. This symptom was combated successfully, and her condition soon gave evidence of convalescence, which was set up in a short time. She improved gradually until the 10th day, when I visited her, leaving her with a very simple prescription, and the injunction that I should be informed of her progress. At this period, she was unfortunately allowed to eat a large slice of bread with butter and honey. This transition from a diet of rice-water was followed by acute pain, and a violent congestive chill, which was succeeded by an exacerbation of fever, lasting the most of the following day. At night her fever entirely subsided, and a period of diaphoresis came on, which lasted for several hours, debilitating the little patient considerably. At this time I was in a dilemma. With a partly subdued acute inflammation, and the periodical element in the disease now making its fearful approach, the administration of some antiperiodic antidote for its neutralization was absolutely and imperiously demanded. At length I resolved to give trial to the sulphate of quinia, combined with all the ipecac the stomach would tolerate, with every attempt to establish an equilibrium in the then embarrassed circulation. The sulphate was exhibited with a liberal hand, but the third day the congestion came again, earlier and in a more aggravated form than before. The evening of that day I visited her shortly before sun-set, and found to every appearane impending dissolution. She seemed to be moribund. The surface was cold and cadaveric; ghastly look of the countenance; a condition of the brain simulating coma, and which seemed to be complete—glassy appearance of the eyes—breathing, sighing and irregular, pulse 165, thready and almost imperceptible.

Viewing the fact that she had a severe rigor that morning, I was brought to a stand for something to do. Recurring to my past treatment, and the critical juncture at which she had arrived, I informed the friends, who had become resigned to her death hours before, that I conceived her case as almost hopeless, as I had expended the energy of the most effective means I had at my command, yet assuring them that it *might* be that she still would recover. I now resolved to try the effect of liberal vinous stimulation, and accordingly a table spoonful of wine was given every two hours, an epispastic fourteen inches long beginning at the occipital protuberance, extending its length down the cervical and dorsal spines: small blisters, too, were ordered to the calves of the legs, and horse-radish leaves to the bottoms of the feet. With the ordering of the foregoing, I took leave, with the strongest injunction that if even the slightest basis took place for further treatment, I should be informed of it. The father called upon me and stated that only after a lapse of twelve hours did the blisters begin to draw, so cold was the surface and so little impression did the large doses of wine produce. But at last the blisters all vesicated freely, and the one over the superior spinal tract relieved the system of at least a quart of serum, which entirely relieved her head symptoms, when she become conscious and smiling, reversing so joyfully the fretful and oblivious state of the mind before mentioned. He said she was better than she had been for days, and instead of the painful forebodings we had all felt, I had the pleasure of visiting her and finding her bolstered up in bed, amusing herself with her toys. I prescribed nothing further of importance, as there was then no indication for anything remedial save wine, which was ordered. A strict hygienic course was also now rigidly enjoined. Next day I found her much improved, and discharged her, and she is now quite well.

I trust that it will not be supposed that I report this, to vaunt my success in the above case; it is not thus. The case was clear, and the indications for treatment lucid, until the supervention of the congestive attacks, which with the existing dregs of sthenic force and action in a vital organ, placed me in a position rather unenviable. The quinine was exhibited in doses sufficiently large, in combination with the ipecac, to produce a decidedly sedative effect. Were the symptoms on the evening when the patient was resigned to the great Destroyer, the result of the powerful sedation produced by remedies, or was that condition an approach to an extinction of the vital dynamics, and relieved by the stimulation, and rapid absorption produced by the generous vesication?

